# Molecular rhythm alterations in prefrontal cortex and nucleus accumbens associated with opioid use disorder

**DOI:** 10.1038/s41398-022-01894-1

**Published:** 2022-03-26

**Authors:** Xiangning Xue, Wei Zong, Jill R. Glausier, Sam-Moon Kim, Micah A. Shelton, BaDoi N. Phan, Chaitanya Srinivasan, Andreas R. Pfenning, George C. Tseng, David A. Lewis, Marianne L. Seney, Ryan W. Logan

**Affiliations:** 1 Department of Biostatistics, University of Pittsburgh, Pittsburgh, PA 15261 USA; 2 Translational Neuroscience Program, Department of Psychiatry, University of Pittsburgh School of Medicine, Pittsburgh, PA 15219 USA; 3 Center for Adolescent Reward, Rhythms, and Sleep, University of Pittsburgh, Pittsburgh, PA 15219 USA; 4 Department of Computational Biology, Carnegie Mellon University, Pittsburgh, PA 15213 USA; 5 Neuroscience Institute, Carnegie Mellon University, Pittsburgh, PA 15213 USA; 6 Department of Pharmacology and Experimental Therapeutics, Boston University School of Medicine, Boston, MA 02118 USA; 7 Center for Systems Neuroscience, Boston University, Boston, MA 02118 USA

**Keywords:** Molecular neuroscience, Addiction

## Abstract

Severe and persistent disruptions to sleep and circadian rhythms are common in people with opioid use disorder (OUD). Preclinical evidence suggests altered molecular rhythms in the brain modulate opioid reward and relapse. However, whether molecular rhythms are disrupted in the brains of people with OUD remained an open question, critical to understanding the role of circadian rhythms in opioid addiction. Using subjects’ times of death as a marker of time of day, we investigated transcriptional rhythms in the brains of subjects with OUD compared to unaffected comparison subjects. We discovered rhythmic transcripts in both the dorsolateral prefrontal cortex (DLPFC) and nucleus accumbens (NAc), key brain areas involved in OUD, that were largely distinct between OUD and unaffected subjects. Fewer rhythmic transcripts were identified in DLPFC of subjects with OUD compared to unaffected subjects, whereas in the NAc, nearly double the number of rhythmic transcripts was identified in subjects with OUD. In NAc of subjects with OUD, rhythmic transcripts peaked either in the evening or near sunrise, and were associated with an opioid, dopamine, and GABAergic neurotransmission. Associations with altered neurotransmission in NAc were further supported by co-expression network analysis which identified OUD-specific modules enriched for transcripts involved in dopamine, GABA, and glutamatergic synaptic functions. Additionally, rhythmic transcripts in DLPFC and NAc of subjects with OUD were enriched for genomic loci associated with sleep-related GWAS traits, including sleep duration and insomnia. Collectively, our findings connect transcriptional rhythm changes in opioidergic, dopaminergic, GABAergic signaling in the human brain to sleep-related traits in opioid addiction.

## Introduction

Despite the enormous public health impact of opioids, understanding of the mechanisms contributing to opioid use disorder (OUD) is limited. Remarkably, ~90% of patients with OUD relapse within 12–36 months of beginning treatment [1]. Identifying the mechanisms that contribute to opioid craving and relapse is critical for developing effective therapeutics and interventions for opioid addiction.

Among the most common features associated with OUD are severe and persistent disruptions to sleep and circadian rhythms (e.g., altered sleep-wake cycles and sleep architecture, poor sleep quality, disrupted corticosterone, and melatonin rhythms), which are speculated to foster craving and relapse [2, 3]. A majority with OUD (~60%) have comorbid sleep and circadian disorders, and many with a history of opioid use and dependence experience poor sleep quality and sleep loss, including insomnia [4, 5]. Further, opioids dose-dependently alter sleep-wake cycles, body temperature, and hormonal rhythms [5, 6]. Notably, sleep and circadian disturbances frequently emerge during opioid withdrawal and accompany intense cravings and negative affective states [7]. With prolonged opioid use, sleep and circadian disruptions become more severe [8–10] and may intensify cravings and mood disturbances [11, 12]. In fact, craving intensity positively correlates with the severity of sleep and circadian disruptions [13, 14]. Therefore, alterations in sleep and circadian rhythms from prolonged opioid use contribute to craving and vulnerability to relapse in people with OUD.

Opioid addiction is also associated with disrupted rhythms in molecular clocks. Molecular clocks are present in nearly every cell in the body, comprised of a series of transcriptional—translational feedback loops primarily driven by the transcription factors CLOCK and BMAL1 [15]. CLOCK and BMAL1 form heterodimers to bind promoters that drive the rhythmic transcription of numerous genes (~40–80% transcripts are rhythmically expressed depending on the tissue and cell type). In the brain, the molecular clock modulates key brain regions that regulate drug reinforcement, craving, and relapse [16, 17]. For example, repeated administration of opioids may entrain molecular rhythms in the brain that ultimately promote drug-seeking and craving at certain times of day [13, 14]. Blunted molecular rhythms in the brain and other tissues are also found following opioid administration in rodents [6, 18, 19]. Similarly, molecular rhythms in circulating lymphocytes are significantly blunted in people with OUD [20]. In addition, certain variants of canonical circadian genes, including *CLOCK*, predict substance use risk and sleep and circadian disruptions associated with substances [21, 22]. Together, these findings suggest bidirectional relationships between opioids and circadian rhythms, whereby opioids alter molecular clocks, ultimately involved in opioid tolerance, craving, and relapse, while disrupted clocks contribute to the overall risk of developing opioid addiction.

From human neuroimaging studies, an increased risk for substance use, including for opioids, is associated with dysfunction of activity in corticostriatal circuits, including signaling between the dorsolateral prefrontal cortex (DLPFC) and nucleus accumbens (NAc) [21, 22]. While the DLPFC is involved in cognitive and emotional control in humans, the NAc is involved in the regulation of goal-directed and reward-seeking behaviors. In OUD, a high degree of dysfunction within the DLPFC and NAc is related to the severity of cognitive impairment and increased relapse risk [23]. Accumulating evidence highlights an integral role for molecular rhythms in opioid-induced synaptic plasticity in corticostriatal circuits involving the DLPFC and NAc [24–27], further supporting the involvement of circadian rhythms in the brain in opioid addiction.

While evidence suggests relationships between altered rhythms and opioid addiction, molecular rhythms have yet to be investigated in the brains of OUD subjects. However, investigating molecular rhythms in the human postmortem brain has historically been challenging. Recently, a series of studies from our group and others have developed an innovative analysis that uses the “time of death” (TOD) of a subject as a single timepoint within a 24 h timescale. By combining data from multiple subjects, we can recreate circadian patterns of transcript expression to investigate alterations in molecular rhythms associated with psychiatric disorders [16,28–31]. To investigate whether molecular rhythms were altered in the brains of subjects with OUD, we directly compared transcript expression patterns using TOD in DLPFC and NAc between OUD and unaffected subjects using our previous dataset [32]. We identified molecular rhythms in both the DLPFC and NAc in unaffected subjects that were significantly altered in OUD. The timing of peak expression of rhythmic transcripts differed between OUD and unaffected subjects and between brain regions. In OUD, altered molecular rhythms were associated with opioidergic, dopaminergic, and GABAergic signaling in the DLPFC and NAc. Finally, we discovered genetic associations between brain-region-specific molecular rhythm changes in OUD and sleep-related traits.

## Materials and methods

### Human subjects

Brains were obtained, following consent from the next-of-kin, during autopsies conducted at the Allegheny County (Pittsburgh, PA; *N* = 39) or the Davidson County (Nashville, TN; *N* = 1) Medical Examiner’s Office. An independent committee of clinicians made consensus, lifetime DSM-IV diagnoses for each subject using the results of a psychological autopsy, including structured interviews with family members and review of medical records, and toxicological and neuropathological reports [33]. The same approach was used to confirm the absence of lifetime psychiatric and neurologic disorders in the unaffected comparison subjects. All procedures were approved by the University of Pittsburgh Committee for Oversight of Research and Clinical Training Involving Decedents and Institutional Review Board for Biomedical Research. Each OUD subject (*n* = 20) was matched with an unaffected comparison subject (*n* = 20) for sex and as closely as possible for age [32] (Tables S1 and S2**)**. Cohorts differed by race (*p* = 0.02) and brain pH (*p* = 0.015; mean difference was 0.2 pH units) and did not differ in postmortem interval (PMI), age, RNA integrity number (RIN), pH, or TOD (*p* > 0.25). DLPFC area 9 and NAc were identified and collected as previously described [32]. Time of death (TOD) was determined from the Medical Examiner’s Office death investigation report.

### Rhythmicity analyses

TOD of each subject was adjusted to circadian time by conversion to Zeitgeber Time (ZT). For each subject, we use sunrise and sunset time on the day the individual died to stratify TOD across ZT. ZT0 is the equivalent of sunrise, where negative numbers represent hours immediately prior to sunrise. Cosinor fitting was used to detect the rhythmicity of transcript expression. Sinusoidal curves were fitted using the nonlinear least-squares method, with the coefficient of determination (*R*^2^) used as a proxy of goodness-of-fit. An estimate of the empirical p-value was determined using a null distribution of *R*^2^ generated from 1000 TOD-randomized expression datasets. Molecular rhythms were first assessed separately in unaffected comparison subjects and subjects with OUD. Rhythmic transcripts were compared using significance cutoffs (*p* < 0.05; Fisher’s exact test to determine overlap) and a threshold-free approach (rank–rank hypergeometric overlap (RRHO)) [34]. RRHO ranks all expressed transcripts (15,042 transcripts in our dataset) by the rhythmicity p-value and determines overlap between the unaffected comparison and OUD datasets based on the ranking of p-values. Transcripts with OUD-related differences in rhythmicity were determined using the difference in *R*^2^. Transcripts with Δ*R*^2^ > 0 when Δ*R*^*2*^ = $R_{{\mathrm{Control}}}^2 - R_{{\mathrm{OUD}}}^2$ were defined as being significantly less rhythmic in OUD. Transcripts with Δ*R*^2^ > 0 when ${\Delta}R^2 = R_{{\mathrm{OUD}}}^2 - R_{{\mathrm{Control}}}^2$ were defined as being significantly more rhythmic in OUD. We generated a null distribution of ${{{\mathrm{{\Delta}}}}}R^2$ by doing permutation 1000 times: at each permutation, we permute the unaffected comparisons and OUD subjects separately with shuffled TOD to get a null $R_{{\mathrm{OUD}}}^2$ and a null $R_{{\mathrm{Control}}}^2$, based on which a null ${{{\mathrm{{\Delta}}}}}R^2$ is calculated. Any transcript with significant Δ*R*^2^ (*p* < 0.05 through permutation test) are denoted as having significantly less or more rhythmicity in OUD. We further restrict the change in rhythmicity analysis to transcripts that are significantly rhythmic in one group or the other. For a transcript to be considered less rhythmic in OUD, it had to: (1) be significantly rhythmic in unaffected comparisons (*p* < 0.05); (2) be significantly less rhythmic in OUD (*p* < 0.05). For a transcript to be more rhythmic in OUD, it had to: (1) be significantly rhythmic in OUD (*p* < 0.05); (2) be significantly more rhythmic in OUD (*p* < 0.05). We also assessed differences in phase, amplitude, or base; analyses were restricted to transcripts significantly rhythmic in both unaffected comparisons and OUD subjects.

### Heatmaps

Transcript expression levels were Z-transformed and ordered by phase (peak hour). Each column represents a subject, ordered by TOD. We generated heatmaps for (1) top 200 rhythmic transcripts in unaffected comparison subjects; (2) top 200 rhythmic transcripts identified in unaffected comparison subjects but plotted for OUD subjects; (3) top 200 rhythmic transcripts identified in OUD subjects; (4) top 200 rhythmic transcripts identified in OUD subjects but plotted for unaffected comparison subjects.

### Scatterplots

Scatterplots were generated to represent transcript expression rhythms. TOD on the ZT scale is indicated on the *x*-axis and transcript expression level on the *y*-axis, with each dot indicating a subject. The red line is the fitted sinusoidal curve. For each brain region, scatterplots were generated for the top three transcripts that were significantly less rhythmic in OUD subjects and the top three transcripts that were significantly more rhythmic in OUD subjects relative to unaffected comparison subjects.

### RNA-sequencing analyses

Overrepresentation of pathways (GO, KEGG, Hallmark, Canonical Pathways, Reactome, BioCarta, CORUM) was assessed using Metascape (http://www.metascape.org) for rhythmic transcripts in DLPFC and NAc, transcripts that were more or less rhythmic in OUD, and transcripts in co-expression networks, with expressed transcripts as background. Networks were visualized with Cytoscape. INGENUITY**®** Pathway Analysis (Qiagen) was used to predict upstream regulators of rhythmic transcripts. Rank–rank hypergeometric overlap (RRHO) [34, 35] was used to assess the overlap of rhythmic transcripts between unaffected comparison subjects and subjects with OUD.

### Identification of OUD-specific co-expression networks

We used weighted gene co-expression network analysis (WGCNA) to identify transcript modules across samples [36]. Networks were built separately in each brain region and disease group. We used Fisher’s exact test to determine enrichment of rhythmic transcripts or transcripts that were significantly less rhythmic or more rhythmic in OUD subjects within each of the WGCNA modules. ARACNe was used to identify hubs for network analysis [37] and Cytoscape was used to visualize networks. Overrepresentation of pathway categories was assessed using Metascape, with 5000 WGNCA-analyzed transcripts as background.

### Integration of rhythmic transcripts with GWAS

Transcripts that were rhythmic within disease groups and transcripts that were less or more rhythmic in OUD subjects (corrected *p* < 0.05) were used to construct foregrounds for GWAS enrichment. We computed the partitioned heritability (GWAS enrichment) of noncoding regions containing and surrounding OUD rhythmic transcript sets using the LD score regression pipeline for enrichment [38]. LD score regression coefficients were adjusted for FDR < 0.05 on enrichments performed on each included GWAS foregrounds. A significant *p*-value indicates enrichment of the foreground genomic regions for GWAS single nucleotide polymorphisms relative to the background.

## Results

### Distinct transcriptional rhythms in subjects with OUD

**Fig. 1:**
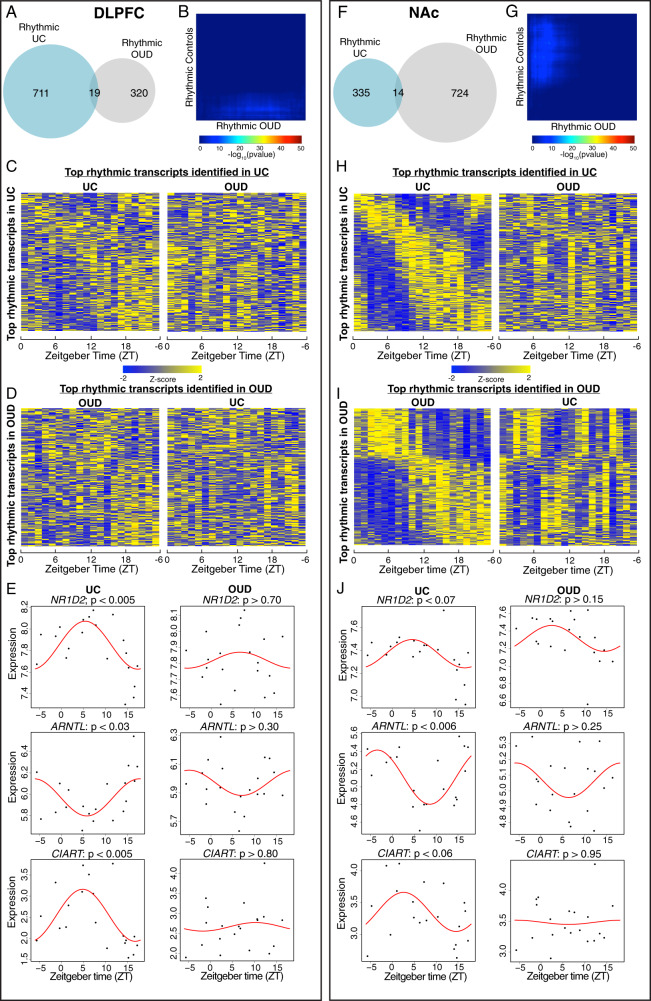
Rhythmic transcripts are largely distinct in unaffected comparison subjects and subjects with opioid use disorder. **A** In the dorsolateral prefrontal cortex (DLPFC), there were 730 rhythmic transcripts detected in unaffected comparison (UC) subjects and 339 in subjects with opioid use disorder (OUD). Notably, only 19 transcripts were rhythmic in both UC subjects and subjects with OUD. Fisher’s exact test indicated lack of overlap in rhythmic transcripts in the DLPFC between UC and OUD subjects (*p* > 0.35). **B** Rank–rank hypergeometric overlap was used as a threshold-free approach to confirm the lack of overlap in rhythmicity patterns in the DLPFC of UC subjects and subjects with OUD. **C** Heatmap of the top 200 circadian transcripts identified in the DLPFC of UC subjects (left), with transcripts peaking across the day. Expression levels are Z-transformed for each transcript, and the transcripts are ordered by their circadian phase value (peak hour). Each column represents a subject and the subjects are ordered by time of death. The top 200 rhythmic transcripts identified in UC subjects are then plotted for subjects with OUD (right), indicating disrupted rhythmicity of normally rhythmic transcripts in subjects with OUD. **D** The top 200 rhythmic transcripts identified in OUD subjects in the DLPFC (left) are then plotted in UC subjects (right). **E** Canonical circadian transcripts (*NR1D2*, *ARNTL*, *CIART*) were rhythmic in the DLPFC of UC subjects, but were not rhythmic in OUD subjects. **F** In the nucleus accumbens (NAc), there were 349 rhythmic transcripts detected in UC subjects and 738 in subjects with OUD. Notably, only 14 transcripts were rhythmic in both UC subjects and subjects with OUD. Fisher’s exact test indicated lack of overlap in rhythmic transcripts in the NAc between UC and OUD subjects (*p* > 0.65). **G** Rank–rank hypergeometric overlap was used as a threshold-free approach to confirm the lack of overlap in rhythmicity patterns in the NAc of UC subjects and subjects with OUD. **H** Heatmap for the top 200 circadian transcripts identified in the NAc of UC subjects (left). The top 200 rhythmic transcripts identified in UC subjects are then plotted for subjects with OUD (right). **I** The top 200 rhythmic transcripts identified in OUD subjects in the NAc (left) are then plotted in UC subjects (right). **J** Canonical circadian transcripts (*NR1D2*, *ARNTL*, *CIART*) were rhythmic in the DLPFC of UC subjects, but were not rhythmic in OUD subjects.

Given the convergence of clinical and preclinical evidence reflecting altered circadian rhythms in opioid addiction [7, 39, 40], we investigated whether brain transcriptional rhythms were altered in DLPFC and NAc, regions strongly implicated in OUD [41–44]. In DLPFC, we identified fewer rhythmic transcripts in subjects with OUD (*n* = 339) compared to unaffected comparison subjects (*n* = 730) (Tables S3 and S4)), with only 19 rhythmic transcripts shared between groups (Fig. 1A**;** Fisher’s exact test *p* > 0.35 indicating nonsignificant overlap). Results using a threshold-free approach, RRHO, further supported a lack of overlap between unaffected and OUD subjects in DLPFC rhythmic transcripts (Fig. 1B). The top 200 rhythmic transcripts peaked across the day in unaffected comparison subjects, but these same transcripts were arrhythmic in OUD subjects (Fig. 1C). Similarly, the top rhythmic transcripts in the DLPFC of OUD subjects were arrhythmic in unaffected comparison subjects (Fig. 1D). Canonical circadian transcripts (*NR1D2*, *ARNTL*, and *CIART*) were rhythmic in the DLPFC of unaffected comparison subjects, like previous studies [16, 28, 29, 31, 45], but were not rhythmic in OUD subjects (Fig. 1E).

We performed a similar analysis of transcriptional rhythmicity in the NAc. Surprisingly, OUD subjects had more than twice as many rhythmic transcripts compared to unaffected comparison subjects (738 and 349, respectively) (Tables S5 and S6), with an overlap of only 14 transcripts (Fig. 1F**;** Fisher’s exact test *p* > 0.65 indicating nonsignificant overlap). RRHO further supported an overall lack of overlap between unaffected comparison and OUD subjects in the NAc (Fig. 1G). In unaffected comparison subjects, the top 200 rhythmic transcripts peaked across the day, while these transcripts were arrhythmic in subjects with OUD (Fig. 1H). Notably, although there are fewer rhythmic transcripts in the NAc relative to DLPFC of unaffected subjects, there is a more robust rhythmicity pattern in the NAc (compare Fig. 1C and H), suggesting that transcripts identified in the NAc are robustly rhythmic. In OUD subjects, the following patterns of diurnal expression were identified in top rhythmic transcripts: (1) peaks of expression during the day and troughs at night; or (2) troughs of expression during the day and peaks at night (Fig. 1I). Using the top 200 rhythmic transcripts in NAc of OUD subjects, these anti-phasic patterns of transcriptional rhythms appear to exhibit near 12 h rhythms of expression in unaffected comparison subjects compared to the 24 h rhythm in subjects with OUD (Fig. 1J). Canonical circadian genes (*NR1D2*, *ARNTL*, and *CIART*) were rhythmic in the NAc of unaffected comparison subjects [30] but were not rhythmic in OUD (Fig. 1J).

### Distinct peak times of rhythmic transcripts in subjects with OUD

**Fig. 2:**
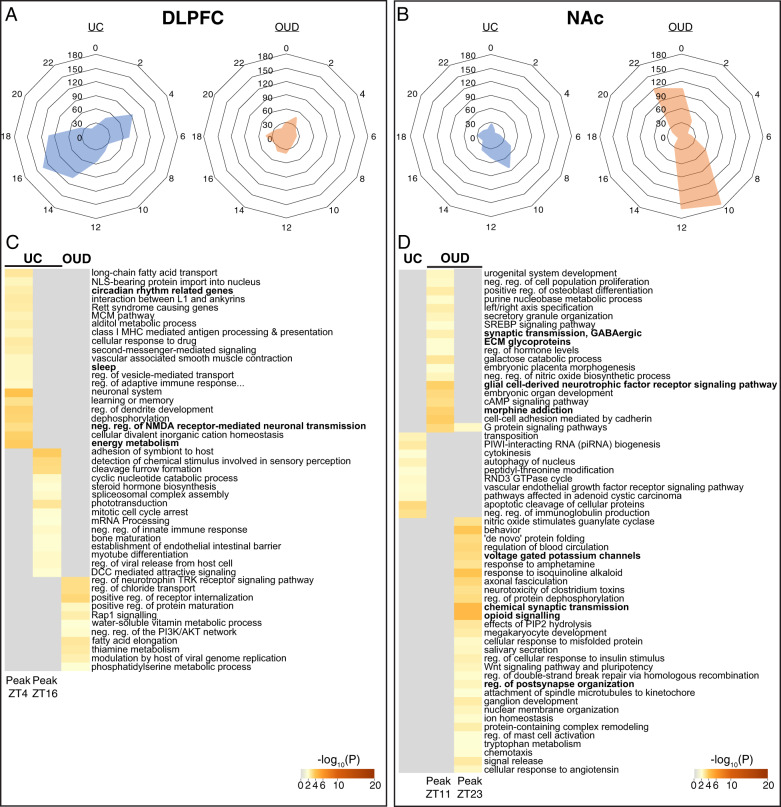
Distinct peak times for rhythmic transcripts in subjects with opioid use disorder (OUD) compared to unaffected comparison (UC) subjects. **A** In the dorsolateral prefrontal cortex of UC subjects, transcripts generally peaked at either ZT4 or ZT16, ~12 h apart. Rhythmic transcripts in the DLPFC in subjects with OUD did not peak at consistent times. **B** In the nucleus accumbens (NAc), rhythmic transcripts in UC subjects generally peaked at ZT10. Rhythmic transcripts in the NAc of subjects with OUD peaked at either ZT11 or ZT23, ~12 h apart. **C** Transcripts peaking at ZT4 in the DLPFC of UC subjects were enriched for pathways related to rhythms (e.g., circadian rhythm-related genes, sleep) and neurotransmission (e.g., negative regulation of NMDA receptor-mediated neuronal transmission), while transcripts peaking at ZT12 were enriched for immune-related pathways (e.g., adhesion of symbiont to host, negative regulation of innate immune response). Rhythmic transcripts in the DLPFC of OUD subjects were enriched for regulation of neurotrophin TRK receptor signaling pathway and positive regulation of receptor internalization. **D** Rhythmic transcripts in the NAc of OUD subjects were enriched for apoptotic cleavage of cellular proteins. In the NAc of OUD subjects, rhythmic transcripts peaking at ZT11 were enriched for morphine addiction, glial cell-derived neurotrophic factor receptor signaling, ECM glycoproteins, and synaptic transmission, GABAergic, while transcripts peaking at ZT23 were enriched for opioid signaling, voltage-gated potassium channels, and synapse-related pathways (e.g., regulation of postsynapse organization, chemical synaptic transmission).

Having identified rhythmic transcripts in the DLPFC and NAc of unaffected comparison and OUD subjects, we next evaluated the timing of peak transcript expression. In DLPFC, we observed two peaks of expression in unaffected comparison subjects at ~ZT4 and ~ZT16 (Fig. 2A, left), nearly 12 h apart from each other; in other words, transcripts tended to peak at either ZT4 or ZT16. In contrast to unaffected comparison subjects, rhythmic transcripts in the DLPFC of OUD subjects did not exhibit distinct expression peaks (Fig. 2A, right). In NAc of unaffected comparison subjects, most rhythmic transcripts peaked ~ZT10 (Fig. 2B, left). Rhythmic transcripts in the NAc of OUD subjects peaked at either ~ZT11 or ~ZT23 (Fig. 2B, right).

The observation that rhythmic transcripts form two separate clusters with peaks ~12 h apart in DLPFC of unaffected subjects and in NAc of OUD subjects prompted further investigation of the biological pathways represented by these clusters of transcripts. In DLPFC of unaffected comparison subjects, transcripts peaking at ZT4 (approximately mid-morning) were enriched for regulation of ion transport, energy metabolism, and negative regulation of NMDA receptor-mediated neuronal transmission, along with circadian rhythm-related genes (Fig. 2C). Transcripts that peak at ZT16 (approximately late evening to midnight) in DLPFC of unaffected comparison subjects were enriched for immune-related pathways (e.g., adhesion of symbiont to host, negative regulation of viral transcription; Fig. 2C). Rhythmic transcripts in the DLPFC of OUD subjects were enriched for pathways related to receptor internalization and neurotrophin signaling (Fig. 2C). In the NAc of unaffected comparison subjects, rhythmic transcripts were enriched for immune pathways (*e.g*., negative regulation of immunoglobulin production) and small noncoding RNAs (e.g., PIWI-interacting (piRNA) RNA biogenesis). Additionally, we investigated pathways associated with rhythmic transcripts peaking at either ~ZT11 or ~ZT23 in NAc of OUD subjects. Interestingly, transcripts peaking at ZT11 and ZT23 were both enriched for opioid-related signaling pathways (Fig. 2D). Transcripts peaking at ~ZT11 (evening) in the NAc of OUD subjects were enriched for morphine addiction, synaptic transmission, GABAergic transmission, and glial cell-derived neurotrophic factor receptor signaling pathways. Transcripts peaking at ~ZT23 (right before sunrise) were enriched for chemical synaptic transmission, voltage-gated potassium channels, and opioid signaling (Fig. 2D). Collectively, these findings indicate rhythmic transcripts: (1) in the DLPFC of unaffected comparison subjects were primarily associated with immune and excitatory synaptic signaling; and (2) in the NAc of OUD subjects were associated with opioidergic signaling and GABAergic neurotransmission.

### Alterations in transcriptional rhythmicity in OUD

**Fig. 3:**
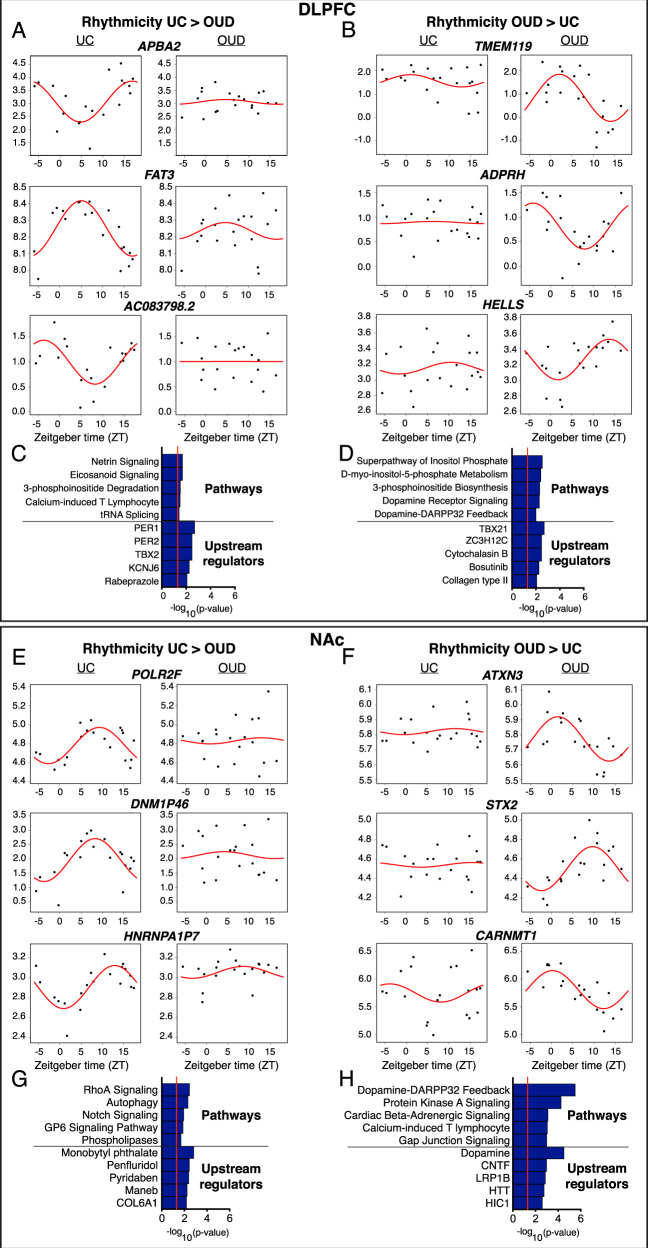
Scatterplots indicating rhythmicity for transcripts that were significantly more or less rhythmic in the dorsolateral prefrontal cortex (DLPFC) and nucleus accumbens (NAc) in subjects with opioid use disorder (OUD) compared to unaffected comparison (UC) subjects. Each dot indicates a subject with *x*-axis indicating the time of death (TOD) on ZT scale (−6 to 18 h) and *y*-axis indicating transcript expression level. The red line is fitted sinusoidal curve. **A** Scatterplots for the DLPFC indicating rhythmicity of *APBA2*, *FAT3*, and *AC083798.2* in UC subjects (left), which are significantly less rhythmic in subjects with OUD (right). **B** Scatterplots for the DLPFC indicating lack of rhythmicity of *TMEM119*, *ADPRH*, and *HELLS* in unaffected comparison subjects (left), and these transcripts are significantly more rhythmic in subjects with OUD (right). **C** The top pathways represented by transcripts that are less rhythmic in the DLPFC in OUD are related to netrin signaling and eicosanoid signaling, and the top IPA-predicted upstream regulators are PER1 and PER2. **D** The top pathways represented by transcripts that are more rhythmic in the DLPFC in OUD are related to inositol and dopamine, and the top IPA-predicted upstream regulators are TBX21 and ZC3H12C. **E** Scatterplots for the NAc indicating rhythmicity of *POLR2F*, *DNM1P46*, and *HNRNPA1P7* in UC subjects (left), which are significantly less rhythmic in subjects with OUD (right). **F** Scatterplots for the NAc indicating lack of rhythmicity of *ATXN3*, *STX2*, and *CARNMT1* in UC subjects (left), and these transcripts are significantly more rhythmic in subjects with OUD (right). **G** The top pathways represented by transcripts that are less rhythmic in the NAc in OUD are related to RhoA signaling and autophagy, and the top IPA-predicted upstream regulators are monobutyl phthalate and the antipsychotic penfluridol. **H** The top pathway represented by transcripts that are more rhythmic in the NAc in OUD is Dopamine-DARPP32 Feedback, and the top IPA-predicted upstream regulators is Dopamine.

Given that we observed minimal overlap of rhythmic transcripts in both unaffected and OUD subjects, we decided to test whether transcripts were significantly less rhythmic or more rhythmic in OUD subjects. In DLPFC, we identified 548 transcripts that were significantly less rhythmic in subjects with OUD relative to unaffected comparison subjects (Table S7). The top transcripts in DLPFC that were less rhythmic in OUD included *APBA2* (amyloid-beta precursor protein-binding family A member 2)*, FAT3* (FAT atypical cadherin 3), and *AC083798.2* (Long noncoding RNA, LncRNA) (Fig. 3A). The top biological pathways associated with transcripts that were less rhythmic in OUD were Netrin and Eicosanoid signaling (Fig. 3C), and the top IPA-predicted upstream regulators included the canonical circadian proteins, PER1 and PER2 (Fig. 3C), suggesting core molecular clock disruptions in DLPFC of OUD subjects.

We also identified 209 transcripts that were significantly more rhythmic in DLPFC of subjects with OUD compared to unaffected comparison subjects, with *TMEM119, ADPRH*, and *HELLS* as the top transcripts (Fig. 3B**;** Table S8). The top pathways associated with transcripts that were more rhythmic in OUD subjects in the DLPFC included inositol biosynthesis and metabolism, along with dopamine receptor signaling and DARPP32 feedback (Fig. 3D), with TBX21 and ZC3H12C as top IPA-predicted upstream regulators.

In NAc, we found 406 transcripts that were significantly more rhythmic in unaffected comparison subjects compared to OUD subjects, including *POLR2F, DNM1P46*, and *HNRNPA1P7* (Fig. 3E; Table S9). From these transcripts, we identified several interacting signaling pathways including RhoA [46], Notch [47], and GP6 [48] (Fig. 3G). IPA-predicted upstream regulators included several pesticide agents with neurotoxic profiles (e.g., monobutyl phthalate) and penfluridol, an antipsychotic medication with primary action at the dopamine 2 receptor [49]. Among the 762 transcripts that were significantly more rhythmic in the NAc of OUD subjects compared to unaffected comparison subjects, the top transcripts included *ATXN3, STX2*, and *CARNMT1* (Fig. 3F; Table S10). Like DLPFC, we identified dopamine-DARPP32 feedback pathways among the top enriched pathways along with dopamine as the top IPA-predicted upstream regulator of transcripts that were more rhythmic in NAc of OUD subjects (Fig. 3H). Other top pathways included protein kinase A, beta-adrenergic, and gap junction signaling, along with the calcium-induced T lymphocyte pathway (Fig. 3H). Overall, our findings suggest pathways related to dopamine signaling were more rhythmic in both DLPFC and NAc of OUD subjects.

### Gene module enrichment of synapse-related and glycoprotein signaling in OUD

WGCNA identified 16 modules in DLPFC of unaffected subjects with only one module enriched for rhythmic transcripts and only one module enriched for transcripts that were significantly less rhythmic in OUD (Fig. S1A). Additionally, we identified 20 modules in DLPFC of OUD subjects; none of these modules were enriched for rhythmic transcripts or for transcripts that were significantly more or less rhythmic in OUD.

**Fig. 4:**
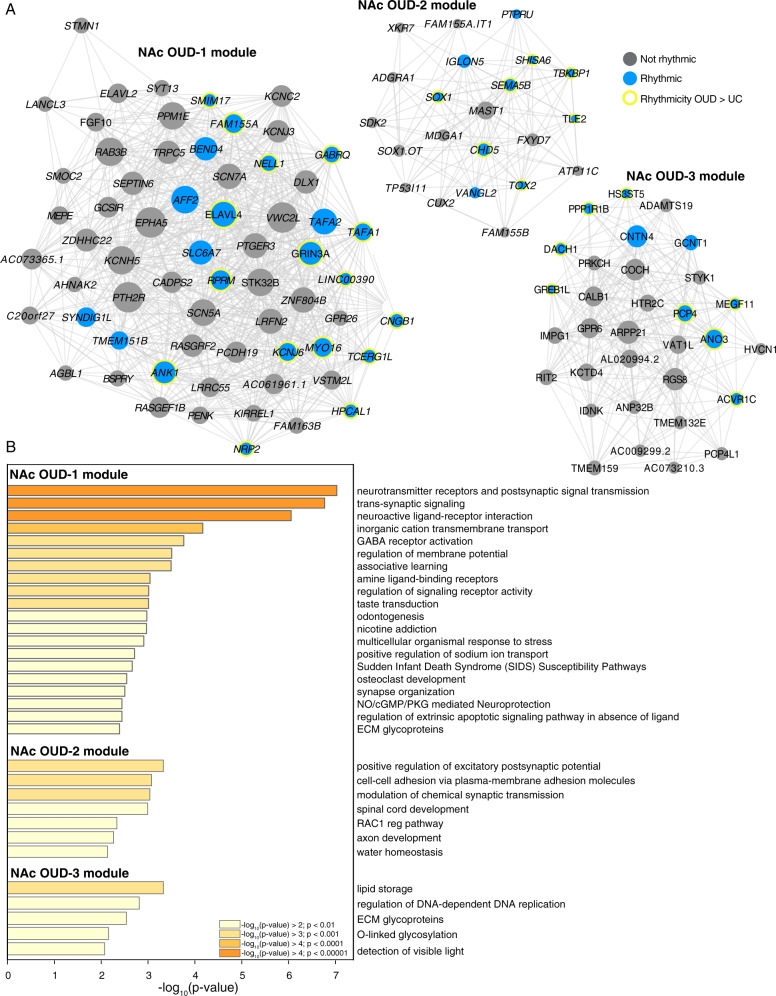
OUD associated gene networks in the NAc. **A** Weighted gene co-expression network analysis (WGCNA) was used to generate co-expression modules, with the network structure generated on each brain region separately. The identified modules that survived module preservation analysis were arbitrarily assigned names and module differential connectivity (MDC) analysis compared the identified modules in OUD and unaffected comparison subjects. **A** MDC analysis indicated a gain of connectivity in the NAc for the OUD-1, OUD-2, and OUD-3 modules. Node size indicates the degree of connectivity for that transcript. Blue nodes indicate rhythmic transcripts and yellow halos indicate transcripts that were significantly more rhythmic in OUD. Edges indicate significant co-expression between two particular transcripts. **B** Pathway enrichment analysis within the NAc OUD-1 module, the NAc OUD-2 module, and the NAc OUD-3 module. Warmer colors indicate increasing −log_10_ *p*-value.

From the 19 modules identified in the NAc of unaffected subjects, only one module was enriched for transcripts that were less rhythmic in the NAc of OUD subjects (Fig. S1B). In OUD, we identified 16 modules in the NAc and three of these modules (OUD-1, OUD-2, and OUD-3) were enriched for rhythmic transcripts. Both OUD-2 and OUD-3 modules were enriched for transcripts that were more rhythmic in the NAc of OUD subjects (Fig. 4A). Several transcripts that are key regulators of synaptic signaling were present in modules OUD-1, OUD-2, and OUD-3. These included: *GRIN3A* [50, 51]*, SLC6A7* [52]*, KCNJ6* [53, 54]*, GABRQ* [55], and *HPCAL1* [56] (OUD-1); *SEMA5B* [57] and *SHISA6* [58] (OUD-2) *PCP4* [59] and *PPP1R1B (DARPP-32)* [60]; (OUD-3). Pathway enrichment analyses of transcripts comprising the networks in the OUD modules further support the connection between rhythmic transcripts in OUD and synaptic function in the NAc, including neurotransmitter receptors and postsynaptic signal transmission, trans-synaptic signaling, positive regulation of excitatory postsynaptic potential, and pathways related to extracellular matrices (ECM) and brain morphology (e.g., ECM glycoproteins, cell-cell adhesion molecules, and axon development) (Fig. 4B).

### Brain-region-specific genetic associations between altered transcriptional rhythms and sleep phenotypes

**Fig. 5:**
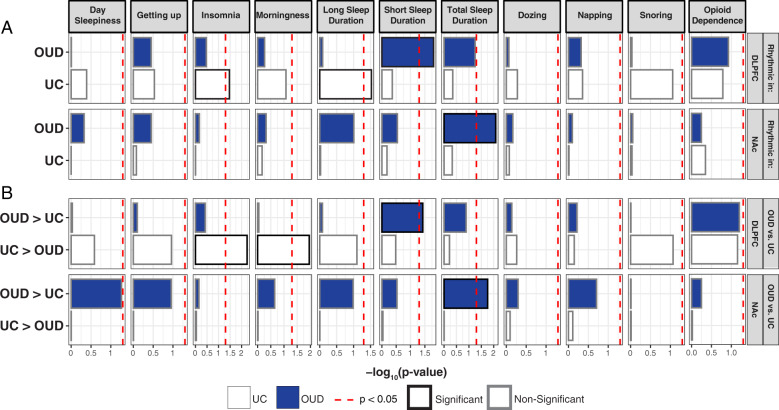
Rhythmic transcripts in the dorsolateral prefrontal cortex (DLPFC) and nucleus accumbens (NAc) enrich for genetic associations with opioid and sleep-related traits. Genome-wide associated studies (GWAS) have identified loci associated with various sleep-related traits and opioid dependence. We investigated whether rhythmic transcripts, as well as transcripts that were significantly more or less rhythmic in subjects with opioid use disorder (OUD), were enriched for genetic associations with sleep-related traits and opioid dependence. **A** In the DLPFC of unaffected comparison (UC) subjects, there was significant enrichment of rhythmic transcripts for genes associated with insomnia and long sleep duration. In NAc of OUD subjects, there was enrichment of rhythmic transcripts and transcripts that were more rhythmic in OUD in total sleep duration. There were no significant associations in the NAc of UC subjects. **B** Insomnia and morningness were associated with transcripts that were significantly less rhythmic in DLPFC of OUD subjects. Transcripts in the DLPFC that were more rhythmic in OUD subjects were enriched for genes associated with short sleep duration, while transcripts that were more rhythmic in the NAc of OUD subjects were enriched for total sleep duration. No significant enrichments were identified for opioid dependence.

Given our findings of altered transcriptional rhythmicity in OUD, we explored whether rhythmic transcripts that are significantly altered in DLPFC or NAc of OUD subjects are associated with opioid and sleep-related traits [61]. To test this idea, we used GWAS studies to integrate significant genomic loci from opioid dependence and various sleep traits (e.g., insomnia, morningness, and sleep duration). Genomic loci identified by GWAS overlap with intronic and distal intergenic noncoding regions within *cis-*acting regulators of gene expression [62]. Using the intronic and distal intergenic regions, we examined whether these genomic regions proximal to transcripts identified as rhythmic either in OUD or unaffected subjects are enriched for genetic associations with opioid dependence and sleep traits. We found no significant associations with rhythmic transcripts in either the DLPFC or NAc with opioid dependence (Fig. 5). However, we identified significant enrichments for rhythmic transcripts in DLPFC of unaffected subjects for insomnia and long sleep duration (Fig. 5A). Transcripts that were more rhythmic in DLPFC of unaffected compared to OUD subjects were significantly enriched in insomnia and morningness (Fig. 5B). In NAc of OUD subjects, highly rhythmic transcripts were significantly enriched for total sleep duration (Fig. 5A), including transcripts that were more rhythmic in OUD compared to unaffected subjects (Fig. 5B). Together, our integrative analyses of rhythmic transcriptomes with human GWAS establishes connections between alterations of transcriptional rhythms in corticostriatal circuitry, opioid addiction, and phenotypes associated with sleep disturbances (i.e., shorter sleep durations and insomnia).

## Discussion

Our data demonstrate transcriptional rhythms in the DLPFC and NAc of unaffected subjects and subjects with OUD. Rhythmic transcripts in unaffected subjects were largely distinct from those in subjects with OUD, suggesting that chronic opioid use leads to the emergence of rhythmicity in specific transcripts involved in the function of corticostriatal circuitry. Indeed, many of the transcripts that were rhythmic in the DLPFC and NAc of OUD subjects were enriched for pathways related to the regulation of dopamine neurotransmission. Using integrative analyses combining circadian patterns of transcriptional regulation and human GWAS, our findings revealed novel gene-trait relationships between transcripts that were significantly more rhythmic in DLPFC and NAc of OUD subjects and sleep-related phenotypes. Additionally, we identified transcripts that were significantly less rhythmic in OUD subjects, also with significant associations to sleep GWAS. An important consideration is that our findings may be driven, in part, by acute opioid administration, as most subjects had a positive toxicology for opioids at the time of death. Since opioid overdose is commonly associated with respiratory failure, an ischemic injury may independently influence brain gene expression. However, many of our unaffected comparison subjects also had causes of death associated with acute ischemia. Future studies using larger cohorts will assess the impact of acute opioid exposure on rhythmic transcript alterations in the human postmortem brain.

Similar to previous studies in the human postmortem brain [30, 32], we found robust transcriptional rhythms in unaffected subjects in both the DLPFC and NAc. In the DLPFC of unaffected subjects, pathways associated with rhythmic transcripts were related to circadian rhythms, sleep, metabolism, immune response, and synaptic and neural transmission. Pathways related to piRNAs, autophagy, and the GTPase cycle were among those enriched in the NAc of unaffected subjects. Notably, there was minimal overlap between the transcripts identified as rhythmic in either the DLPFC or NAc from unaffected subjects compared to OUD subjects. Several pathways, which have been previously linked to the effects of opioids, were enriched in rhythmic transcripts in the DLPFC of OUD subjects, including neurotrophin TRK receptor signaling [63, 64] and Rap1 signaling [65]. For example, neurotrophin activation of TRK receptors, signaling through various molecular cascades (e.g., cAMP and ERK), is involved in opioid-induced neural and synaptic plasticity [66, 67]. Dysfunction in neurotrophin TRK receptor signaling and opioid receptor signaling has been associated with psychiatric disorders [64]. Moreover, Rap1 may be involved in a subfamily of GTPase-activating proteins that influence mu-opioid receptor activation [68] and neurotransmission [65]. Interestingly, Rap1-dependent signaling modulates neuronal excitability and drug reward-related behaviors in mice [69].

In the NAc of OUD subjects, enriched pathways from rhythmic transcripts were related to GABAergic neurotransmission, morphine, opioid signaling, postsynaptic organization, and glial cell neurotrophic factors, along with ECM glycoproteins, among others. Interestingly, we recently reported that transcripts associated with microglial and ECM pathways were differentially expressed in DLPFC and NAc of OUD subjects when the time of death was not taken into consideration [32]. The current results suggest these transcripts and their related pathways may be altered at specific times of the day. While the functional impact of rhythmic alterations in glia [70] and brain scaffolding [71, 72] needs to be explored further in OUD, microglial regulation of neuroinflammation, in addition to consequences on the ECM and the functional impacts on synaptic physiology, may be critically involved in the long-term effects of opioids on the brain [32, 73, 74].

Many of the rhythmic transcripts we identified in the DLPFC of unaffected subjects and in the NAc of OUD subjects generally peaked at different times of the day. In unaffected subjects, rhythmic transcripts tended to peak at either ZT4 (i.e., mid-morning) or ZT16 (i.e., late evening), represented by distinct sets of enriched pathways. For example, circadian rhythm and sleep-related transcripts peak at ZT4, while other pathways peak at ZT16. In OUD subjects, rhythmic transcripts did not exhibit these two peaks, possibly due to this group having fewer than half the rhythmic transcripts compared to the unaffected group. In contrast, rhythmic transcripts in the NAc exhibited two peaks at ZT11 (i.e., evening) or ZT23 (i.e., prior to “sunrise”) in OUD subjects. Several of the pathways peaking at ZT11 were related to glia, ECM, GABAergic signaling, and morphine addiction, while the pathways peaking at ZT23 were potassium channels, synaptic transmission, opioid, and Wnt signaling, and others. We also observed hints that transcripts exhibiting a 24 h rhythm in the NAc of OUD subjects might exhibit ultradian rhythms (i.e., less than 24 h) in unaffected comparison subjects, although we were not powered to determine if these transcripts did indeed exhibit 12 h rhythms. Twelve hours rhythms in neuronal and synaptic activity, neurotransmission (e.g., dopaminergic [75]), and behaviors [16,27,28] have been described in rodent models [76].

In subjects with OUD, we found transcripts that were significantly less rhythmic in DLPFC and NAc compared to unaffected comparison subjects; these transcripts were related to many pathways of brain function, including synapse and immune signaling. For example, the transcript *APBA2* was less rhythmic in the DLPFC of OUD subjects and encodes for a synaptic adaptor protein, which when disrupted leads to the impaired synaptic formation and vesicle trafficking in excitatory synapses [77]. In addition, *APBA2* variants were associated with impulsivity and addiction vulnerability [78]. APBA2 directly binds neurexin proteins that are neuron-specific surface proteins involved in synaptic formation and netrin signaling [79]. Netrin signaling was the top pathway enriched from transcripts that were less rhythmic in the DLPFC of OUD subjects. Other pathways included eicosanoid signaling, involved in synaptic plasticity and inflammation [80]; 3-phosphoinositide degradation, involved in neuronal hyperexcitability and associated with various psychiatric disorders [81]; calcium-induced T lymphocyte, which tunes T-cells to coordinate immune responses [82]; and tRNA splicing [83]. In the NAc of OUD subjects, transcripts that were less rhythmic were related to synapses and substance use. For example, *HNRNPA1P7* belongs to a family of RNA-binding proteins involved in cytoskeletal organization and synaptic activity, and more recently, substance use [84]. Both RhoA and Notch signaling pathways were also enriched for transcripts that were less rhythmic in NAc of OUD subjects, both of which are involved in opioid tolerance and withdrawal [46].

Additionally, we identified transcripts that were significantly more rhythmic in OUD subjects. For example, *TMEM119*, a robust marker for microglia [85], was among the top transcripts that were highly rhythmic in the DLPFC of OUD subjects, resembling increased glial reactivity at certain times of day [86] in OUD. In support of this, *Tmem119* has a robust expression rhythm in the mouse suprachiasmatic nucleus associating with circadian-dependent modulation of glial activity [87]. Many of the top pathways enriched among transcripts that were more rhythmic in DLPFC of OUD subjects were related to inositol phosphates, key regulators of cell signaling [88]. The inositol phosphate pathway impacts cellular and molecular rhythms [89], suggesting molecular changes in diurnal patterns of expression in the DLPFC of OUD subjects are, in part, driven by alterations in inositol signaling. Other pathways included feedback regulation of dopamine neurotransmission, and the top IPA-predicted upstream regulators were TBX21 and ZC3H12C; both of these transcription factors modulate dopamine’s actions on immune cells in the brain, controlling the activation of T-cells, consequently regulating the neuroinflammatory response [90].

In contrast to DLPFC, most transcripts that were significantly more rhythmic in the NAc of OUD subjects were related to synapses. For instance, *ATXN3*, among the top rhythmic transcripts, is involved in the formation of dendritic spines and new synapses in rodents [91] and *STX2* regulates vesicle release of neurotransmitters [92, 93]. In addition, *GRIN3A* was significantly more rhythmic in NAc of OUD subjects, and notably, was identified as a hub transcript in the OUD-1 module. The OUD-1 module was specific to OUD and the NAc, and was mainly comprised of transcripts involved in neurotransmission, such as postsynaptic receptors, trans-synaptic signaling, neuroactive ligand-receptor signaling, and GABA receptor activation. *GRIN3A* is involved in opioid-induced synaptic plasticity of both excitatory and inhibitory circuits in the NAc following chronic administration [51, 94, 95] and variants of *GRIN3A* were identified as alleles associated with therapeutic response to methadone in people with opioid addiction [96]. Previously, we found that neuroinflammatory pathways were enriched in differentially expressed transcripts in the NAc from OUD subjects, including interferon (IFN) signaling [32]. Intriguingly, IFN signaling interacts with GRIN3A, whereby elevated IFN induces NMDA-evoked glutamate release [50]. Thus, cycles of opioid withdrawal may elevate IFN levels, augmenting excitatory signaling in NAc and inducing opioid-induced synaptic plasticity and behavioral consequences. Based on our findings, rhythmicity of GRIN3A-dependent signaling may also regulate opioid-induced excitatory synaptic plasticity [97].

We integrated the transcriptional rhythm profiles in the human brain with opioid and sleep-related GWAS findings to begin to identify novel gene-trait relationships in OUD. Using integrative GWAS analyses, we found that transcripts that were less rhythmic in the DLPFC of OUD subjects were enriched for genomic loci associated with insomnia and morning preference GWAS. Further, transcripts that were more rhythmic in DLPFC of OUD subjects were associated with short sleep duration GWAS. Transcripts that increased transcriptional rhythmicity in the NAc of OUD subjects were related to total sleep duration. We found a lack of enrichments for opioid-related GWAS, probably because of the comparatively smaller sample sizes and limited GWAS loci for opioid dependence [61]. Nevertheless, our findings support associations between genetic risk for sleep alterations, brain-region-specific changes in transcriptional rhythmicity, and OUD. Given the roles for both the DLPFC [98] and NAc [99–101] in sleep and substance use, our results provide putative mechanisms for transcriptional rhythmicity in DLPFC and NAc to mediate the relationships between sleep and OUD. Our findings provide further support for the involvement of dopaminergic and glutamatergic neurotransmission in corticostriatal circuits including the DLPFC and NAc in the regulation of sleep and possible intersections with substance use [102]. Further, our results suggest treatments targeting certain pathways in the brains of patients with OUD may be more effective when given at the time of day when the alteration is most robust. Our insights will hopefully provide the opportunity for new therapeutics in the treatment of OUD.

## Supplementary information

Supplemental Material

Supplemental Material
